# Towards perturbation prediction of biological networks using deep learning

**DOI:** 10.1038/s41598-019-48391-y

**Published:** 2019-08-16

**Authors:** Diya Li, Jianxi Gao

**Affiliations:** Rensselaer Polytechnic Institute, Department of Computer Science, Troy, 12180 USA

**Keywords:** Statistical physics, thermodynamics and nonlinear dynamics, Computer science, Computational science

## Abstract

The mapping of the physical interactions between biochemical entities enables quantitative analysis of dynamic biological living systems. While developing a precise dynamical model on biological entity interaction is still challenging due to the limitation of kinetic parameter detection of the underlying biological system. This challenge promotes the needs of topology-based models to predict biochemical perturbation patterns. Pure topology-based model, however, is limited on the scale and heterogeneity of biological networks. Here we propose a learning based model that adopts graph convolutional networks to learn the implicit perturbation pattern factors and thus enhance the perturbation pattern prediction on the basic topology model. Our experimental studies on 87 biological models show an average of 73% accuracy on perturbation pattern prediction and outperforms the best topology-based model by 7%, indicating that the graph-driven neural network model is robust and beneficial for accurate prediction of the perturbation spread modeling and giving an inspiration of the implementation of the deep neural networks on biological network modeling.

## Introduction

**Figure 1:**
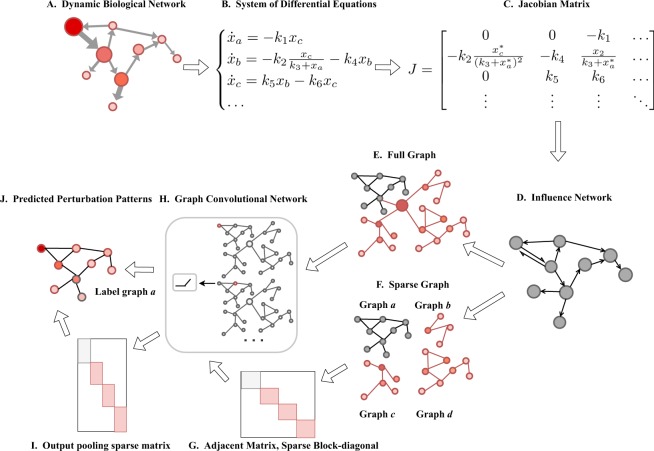
Flowchart of the enhancement method based on graph convolutional networks. (**A**) The topological of a directed biological network. (**B**) The dynamics of system in *A* can be described by a set of nonlinear differential equations. (**C**) The Jacobian matrix of the differential equations in (**B**) at its steady state. (**D**) The influence network topology can be mapped out from the Jacobian matrix in (**C**) corresponding to its adjacent matrix. (**E**) We gather all the influence networks together and generate the full graph. (**F**–**J**) Perturbation prediction using graph convolutional networks.

Nowadays, the development of high technologies makes it possible to map a large portion of biochemical entity interactions into one component. However, while the discovery of interactome keeps increasing, it is hard to measure the information loss by the limitation of kinetic parameter measurement which reflecting the dynamics of biochemical entity interactions. One practical way to measure all interactions without acquiring kinetic parameter information is using the biological networks. Figure 1A demonstrates a dynamic biological network, where a node denotes a biological species or disease and a link represents the interaction between a pair of them. In order to understand the underlying dynamics of this system, we have to predict the response of it under different perturbations, showing how the perturbation spreads in the network^1–5^. For instance, the red node is the perturbation source as shown in Fig. 1A, and the degrees of red color in other nodes demonstrate the propagation states of such perturbation in this biological network. Prediction of perturbation pattern is often used to measure the influence among species in a biological system for the given source. It helps us determine which species play important roles so that small perturbation on them may cause dramatic changes of other nodes and how they interact with each other. Finding such perturbation pattern is crucial for biology community to understand the differential expression patterns discovered in species states while the quantitative dynamical framework is still scarce for such pattern prediction modeling partly because of the rarity and difficulty of large-scale measurements of kinetic parameters in biology model^6,7^. To overcome the limitation of scarce kinetic parameters, some studies^8,9^ have proposed models to retrieve global perturbation properties from the minor models^9^ or the most probable dynamical model from perturbation statistics in reverse^8^. However, such global measures are limited to predicting small size biological models with heterogeneous kinetic parameters. Methods of adopting pure network topology knowledge to make perturbation pattern prediction, such as Boolean networks^10^ and normalized-Hill models (NHMs)^11^ could achieve promising accuracy on a few well-described, small networks, but are not practical on diverse real-world biological networks. Previous studies^11,12^ reveal that the dynamics in biological systems is mainly determined by the network topology, but not by detailed kinetic parameters. Santolini Marc and Barabási Albert-László^5^ proposed a series of network topology based model named “DYNamics-Agnostic Network MOdels (DYNAMO)”, which can retrieve the relative magnitude of biological perturbation patterns when lacking the knowledge of the kinetic parameters. The DYNAMO model series achieve an average of 65% accuracy on perturbation pattern predicting of the full biochemical model and a simple distance-based model in this series shows promising results. We ask the question: how to improve the perturbation pattern prediction of the only network topology-based method by deep learning?

Deep neural networks have broad applications in the biological domain and show effectiveness on several biological learning tasks^13–15^. Among them, graph neural networks^16–19^ are designed for learning tasks on graph data (i.e., citation networks, knowledge graphs, protein-interaction networks) by capturing the dependence of graphs by passing messages among graph nodes^20^. As a variant of graph neural network, the graph convolutional networks (GCN)^16^ learn hidden layer representations by encoding both local graph structure and nodes features via spectral graph convolutions.

As previous methods focus on pure topology information of the given biological network without exploring the information from external biological networks, here we adopt the GCN model to jointly learn the prediction pattern from large scale external biological networks. We find that the rank of influence among species in the biological network has a crucial impact on perturbation pattern and could further enhance the distance model in DYNAMO. We call such influence rank relation in the biological network as an impact pattern. To learn such impact pattern from a large set of biological networks, we consider the scenario as a node classification problem in a graph where the impact factor label of each node is only available for a small subset of nodes in the graph. Graph convolutional network (GCN) encodes graph structure of a biological network directly and learns representations of the graph nodes both with and without impact factor labels. In this paper, we adopt the GCN structure and unite all biological networks as a giant graph as the input of this neural network and then get the predicted impact factor of unlabeled nodes after training and test processes.

After getting the impact pattern which is a weak rank matrix among species, we set the impact pattern as an enhancement factor multiplied to another rank matrix regarding the distance of species in the biological network to enhance the final perturbation pattern prediction. The intuition here is that we find using network topology only is not enough for accurate perturbation pattern prediction. Thus, we incorporate GCN to learn an enhancement factor (a rank matrix among species) to enhance the topology-based model. Using the same dataset as DYNAMO used, we evaluate our model on 87 biological networks and the results show 73% accuracy on perturbation pattern prediction.

## Methods and Materials

### DYNAMO model and perturbation pattern prediction

A biological network is a framework to describe and understand cellular processes and the mechanism of perturbation affecting disease states^21–25^. The biological network helps biology community to quantify and predict the spread of perturbations across the network in terms of the interactome. Discovering perturbation patterns requires the construction of dynamic models, but the difficulty is rooted in the limited knowledge acquisition of the kinetic parameters. To overcome these problems, Santolini Marc and Barabási Albert-László^5^ proposed DYNAMO (DYNamics-Agnostic Network MOdels). They got the Jacobian matrix from the differential equations of a biological model. The Jacobian matrix is used to construct the underlying weighted topology of the biological models and get the influence network within one biological model. Note that one can build the full biochemical model from the Jacobian matrix, which contains the entire kinetic parameters information. As shown in Fig. 1A–D, we get the system differential equations from the dynamic biological network, and obtain the Jacobian matrix when the system is around its steady state. Then we can get the influence network and the full biochemical model from the Jacobian matrices. The full biochemical model defines the ground truth for the evaluation of DYNAMO.

Given an influence network, the goal of perturbation pattern prediction is to predict how the perturbation of a species propagates over the network to other species and to what degree it affects other species. We can use the sensitivity matrix *S*_*ij*_ to represent such perturbation patterns. The sensitivity matrix describes the change in the steady-state value *x*_*i*_ of a node *i* when the steady-state value *x*_*j*_ of another node *j* is varied, computed as ${S}_{ij}=\frac{d{x}_{i}}{d{x}_{j}}$. Three models in DYNAMO have been proposed to generate sensitivity matrices using network topologies, they are the propagation model, the distance model, and the first neighbor model. In the propagation model, the predicted perturbation of a node is proportional to the degree-weighted sum of the perturbations of its neighbors. A simpler model, the distance model assumes that the strength of a perturbation is inversely proportional to the network distance between a species and the source of perturbation. The simplest model, the first-neighbor model assumes that the perturbation reaches only the direct neighbors of a perturbed node. The Spearman rank correlation method^26^ is used to compare the correlation between the predicted sensitivity matrix and the ground truth sensitivity matrix which is derived from the original biological system with full kinetic parameters. The Spearman rank correlation can be calculated as:

1 $$\rho =\frac{cov(r{g}_{X},r{g}_{Y})}{{\sigma }_{r{g}_{X}}{\sigma }_{r{g}_{Y}}}$$

where *rg*_*X*_, *rg*_*Y*_ are the predicted sensitivity matrix and the ground truth ranked by column, *cov*(*rg*_*X*_, *rg*_*Y*_) is the covariance of the rank variables and ${\sigma }_{r{g}_{X}}{\sigma }_{r{g}_{Y}}$ are the standard deviations of the rank variables. The rank correlation score represents the accuracy of the prediction. In DYNAMO series, the propagation model achieves 66% of accuracy when the network includes direction and sign of the links. Surprisingly, a simple distance model on directed network achieves 63% accuracy. The simplest first neighbor model only achieves up to 27% accuracy.

### Enhancement factor design

The DYNAMO uses pure network topology knowledge and achieves promising prediction accuracies. Instead of only adopting network topology, we also want to leverage the features in full biochemical model into network topology thus to enhance the perturbation prediction. We observe that leveraging the rank information in the full biochemical model and setting it as an enhancement factor on the distance model could significantly improve the original model.

Thus, we have designed a novel method to generate the enhancement factor *E* which could be learned from the full biochemical model. The method to generate the enhancement factor describes as follows:

- For a given biomodel, we get a **bio-impact matrix** by ranking the values in each column in its Jacobian matrix. Here we ignore the impacts among perturbation sources.
- After getting the bio-impact matrix, we notice that the range of the rank values is too broad for training a generalized graph convolutional network for prediction because the rank value could be greater than 200 if the biomodel contains more than 200 species. Thus, we scale down the rank values using a *log* operation, $scaled\_rank=\lceil {\mathrm{log}}_{2}(rank)\rceil +1$. We denote the scale down matrix as **scaled impact matrix**.
- We normalize the scaled impact matrix by dividing each column with its corresponding diagonal element. The final **normalized scaled-matrix** is our enhancement factor *E*.

Take the biomodel BIOMD0000000168 in BioModel database^27^ for example, it contains nine species but only seven species (*D*_1, *E*_1, *RS*_1, *R*_1, *X*_1, *E*2*F*_1, *RP*_1) get involved in dynamic interactions. The Jacobian matrix of this model is:

The bio-impact matrix is:

The down scaled impact matrix is:

Finally, the normalized scaled impact matrix also the enhancement factor *E* is:

### Enhancement factor prediction via graph convolutional networks

To improve the pure network model, we design an enhancement factor *E* from the full biological model. With the generated enhancement factor *E* as the gold label of the network, we use the graph convolutional network to predict the enhancement factors on other networks with setting a large portion of networks as the training dataset. The graph convolutional network^16^ aims to solve the problem of classifying nodes in a network where labels are available for a subset of nodes. The so-called graph convolutional network is different from the “classic” convolution neural network^28^ as it deals with graph-structured data and shares filter parameters over all locations in the graph^29^.

**Figure 2:**
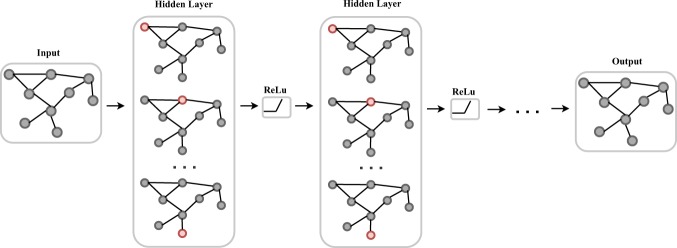
Graph convolutional networks.

The input of a graph convolutional network is an *N* × *D* feature matrix *X* in which *N* is the number of nodes and *D* is the number of input features and representative description of the graph structure, typically choosing the adjacency matrix *A* of the graph. The output is an *N* × *F* feature matrix *Z*, where *F* is the number of output features per node. Every neural network layer could be written as a non-linear function:

2 $${H}^{(l+1)}=f({H}^{(l)},A)$$

where *H*(0) = *X* and *H*(*L*) = *Z*, *L* is the number of neural network layers. Given the definition of each neural network layer, a simple form of a layer-wise propagation rule could be written as:

3 $$f({H}^{(l)},A)=\sigma ({\hat{D}}^{-\frac{1}{2}}\hat{A}{\hat{D}}^{-\frac{1}{2}}{H}^{(l)}{W}^{(l)})$$

where *W*^(*l*)^ is a weight matrix for the first neural network layer; $\hat{A}=A+I$, *I* is the identity matrix. $\hat{D}$ is the diagonal node degree matrix of $\hat{A}$, $\sigma (\cdot )$ is a non-linear activation function, here we use ReLU^30^. The structure of the graph convolutional network is shown in Fig. 2.

To leverage the graph convolutional network for enhancement factor prediction, we propose two models: (1) **full graph model** and (2) **sparse graph model**.

- **full graph model**: we gather all the influence networks together with an extra center node linking to other nodes which has the largest degree in each influence network. We denote such procedure as graph data preprocessing, as shown in Fig. 1E,F.
  Taking the new giant network as the input graph, the output of the graph convolutional network is the feature matrix with *N* columns, and each feature is the enhancement label per node as demonstrated in Fig. 1H,J.
- **sparse graph model**: different from the full graph model, the input of the graph convolutional network is the concatenation of respective feature matrices of influence networks. The adjacent matrix of the network input is a sparse block-diagonal matrix where each block corresponds to the adjacency matrix of each influence network. The adjacent matrix is shown in Fig. 1G.

For the sparse graph model, the pooling operation of graph convolutional network requires a specified pooling matrix which collects features from their respective graphs. Figure 1I illustrates the pooling operation. The output of the sparse graph model is the same as the full graph model.

### Dataset and experimental setup

Following previous work^5^, we test our method on biological models from the BioModels database^27^ which is a repository of curated biological dynamic models. To get the training dataset, we use the BioModels_Database-r30_pub-sbml_files. There are 611 curated BioModels in SBML file format, we use the **libsbml** matlab library^31^ to extract the differential equations describing dynamics from the SBML file and we successfully map out 333 BioModels as our training and test datasets.

To train the graph convolutional network, we implement the model with the usage of the Pytorch library. We optimize the cross-entropy loss as objective function by using Adam^32^. To avoid overfitting, we add a dropout layer with the dropout probability of 0.5. We set the initial learning rate as 0.01. We train for up to 200 epochs and use the final model to predict the test dataset.

## Results

### Perturbation pattern prediction analysis

**Figure 3:**
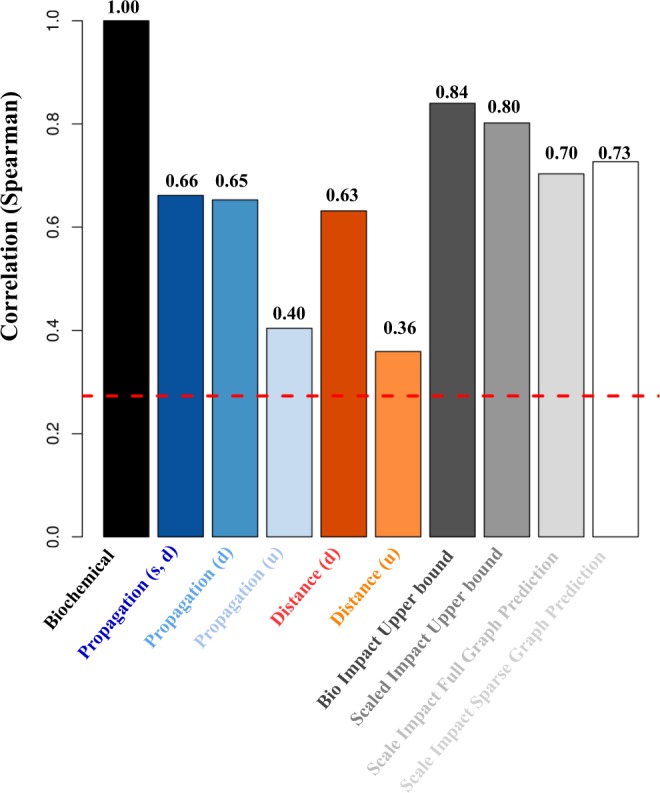
Overall results on perturbation pattern prediction.

The perturbation pattern predictions are shown in Fig. 3 and the exact numbers are listed in Table 1. The black bar is the full biochemical model which is the ground truth in our experiment, the blue bars and red bars are the results on propagation and distance models corresponding to DYNAMO respectively. The grey bars are the upper bounds of our proposed enhancement models and the experimental results on graph convolutional networks. The first grey bar shows the upper bound of using full impact matrix onto the distance model, which is 84% in our case. It indicates that if we get the 100% accuracy on enhancement factor prediction on graph convolutional networks, we will achieve 84% accuracy on perturbation pattern prediction. The second grey bar shows the upper bound of using normalized and scaled impact matrix onto the distance model, and we get an upper bound in 80% accuracy. In the rightmost two columns, the light grey bar and white grey bar demonstrate the scaled impact matrix prediction respectively using full graph model and sparse graph model enhanced in distance model, and the prediction accuracies are 70% and 73% in our experiment. We gain 7% and 10% improvements on perturbation pattern prediction accordingly comparing to the original distance model (63%). We can see that the sparse graph model outperforms the full graph model as most biological networks are heterogeneous. The most significant difference between sparse and full graph model is that the former has a specific pooling matrix according to the feature matrix of each biological network.

**Table 1:** Overall results on perturbation pattern prediction, 0.66 is the best performance of DYNAMO, 0.63 is the accuracy of distance model.

	Model Name	Correlation Score
**Ground truth**	Biochemical	1.00
*DYNAMO^5^	Propagation (signed, directed)	**0.66**
	Propagation (directed)	0.65
	Propagation (undirected)	0.40
	Distance (directed)	**0.63**
	Distance (undirected)	0.36
*Our model	Upper Bound of Bio_Impact	0.84
	Upper Bound of Scaled_Impact	0.80
	Scaled_Impact Full Graph Prediction	**0.70**
	Scaled_Impact Sparse Graph Prediction	**0.73**

0.70 and 0.73 are the accuracies of our proposed enhancement methods on distance model.

Using the sparse graph model, we get a 7% accuracy increment comparing with the best propagation model (66%) on a directed signed network in DYNAMO. An interesting observation is the gap between bio-impact upper bound and the scaled-impact upper bound is not huge. The scaled impact matrix prediction is more adaptable to train a generalized graph convolutional network. Thus, with the sacrifice of a higher upper bound, the easier scaled impact matrix prediction task is preferred in our model and the results indicate that our approach achieves excellent prediction of the perturbation patterns.

**Figure 4:**
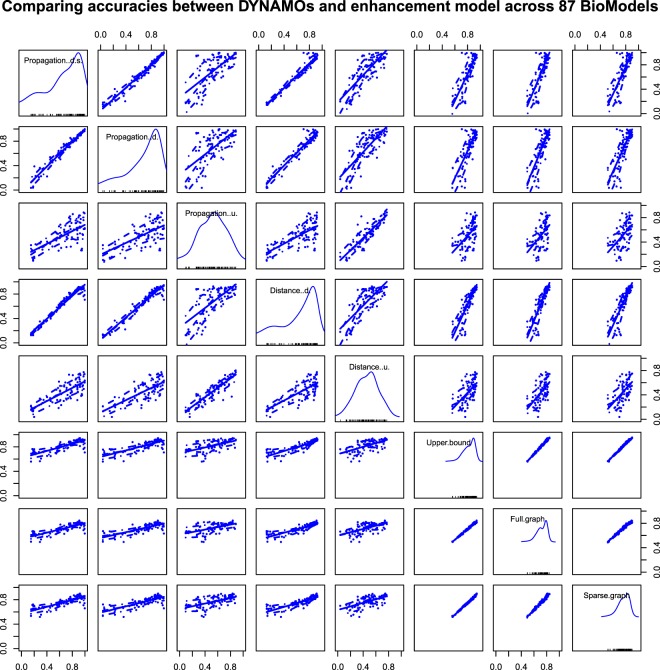
Pairwise accuracy between DYNAMO and enhancement models on the selected 87 Bio-Models.

Figure 4 visualizes the pairwise accuracies between DYNAMO and our enhancement models. In this figure, the diagonal subfigures show the distribution of accuracies from each model, and the off-diagonal subfigures represent scatterplots of one model versus another. We can see from Fig. 4 that the model from the same series is highly correlated. For example, our enhancement models which shown in the last three columns and rows are derived from the same graph convolutional networks and intuitively have similar performances on the 87 BioModels. Comparing to the accuracy distribution on an individual model shown in the diagonal subfigures, we find that the enhancement models (full and sparse graph models) even achieve relatively high prediction accuracies when the overall prediction is low in some BioModels which means the enhancement models are more stable and robust than DYNAMO series.

**Figure 5:**
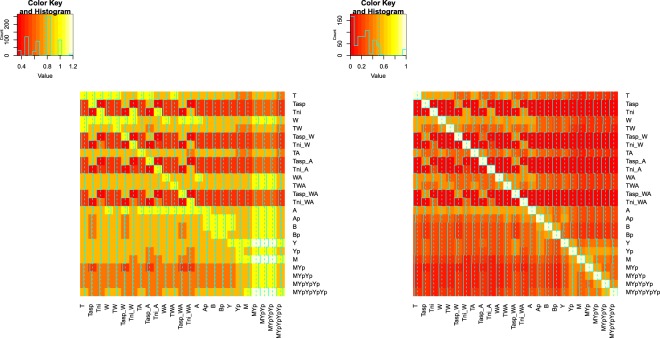
Heatmaps of the BioModel (left) and sparse graph model (right) on **BIOMD0000000404**.

To visualize the correlation of the ground truth and our predicted results, we plot the heatmaps of perturbation pattern predictions via BioModel and our sparse graph model. As shown in Fig. 5, the color in the heatmap represents the perturbation influence between two species. The lighter the color is, the greater of the influence. As shown in Fig. 5, the color distributions are similar in the two heatmaps which means our learning method based model can make promising predictions comparing to the ground truth BioModel with full kinetic parameters.

### Network property analysis

**Figure 6:**
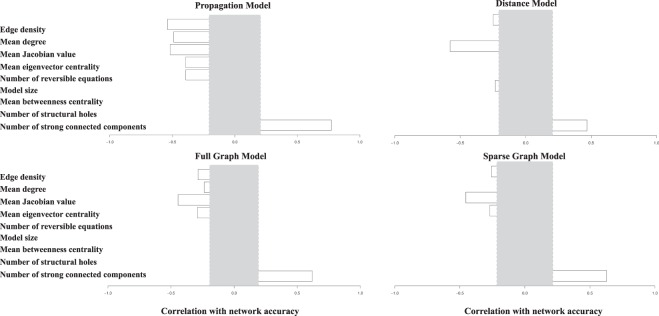
Correlation between properties (names on the left) and the propagation, distance and enhancement models (full graph model and sparse graph model) accuracies across 87 BioModels. Grey areas are the random expectations.

Figuring out the network properties which are crucial for the model’s prediction performance is beneficial for result analysis and future investigation. We’ll have a first glance of the prediction performance by looking at the network properties that are essential attributes. To investigate the effect of network properties on perturbation pattern prediction, we show the correlation between network properties and different models’ accuracies in Fig. 6. We list the selected property names on the left and show the random expectations in grey areas. The random expectation is the correlation span which calculated by computing the standard deviation of different correlation scores between prediction accuracy and randomly generalized values. Among them, model size denotes the number of nodes in the network, mean Jacobian value is from the log_10_ of the original value. The number of structural holes means node Burt’s constraint (low constraint corresponding to many “structural holes”). As shown in Fig. 6, the number of strongly connected components has a positive effect on all the models while the mean Jacobian value always performs a negative role in perturbation pattern prediction which indicates the larger the Jacobian value is, the worse the model’s performance.

We also notice that network properties have many constraints in the propagation model while having fewer effects in distance models including our enhancement models. It is reasonable as the propagation model highly relies on the network topology while our enhancement model partially relies on the learning model. We can also see that the sparse graph model is more independent than the full graph model as it deals with individual networks in graph convolutional networks while the full graph model must consider the whole network. Overall, network properties have fewer constraints in our proposed learning-based model, indicating the generality and portability of our model. We can further apply our model to other dynamic networks with less constraint of the individual network properties.

## Discussion

Perturbation pattern prediction on biological networks is crucial for the biology community to study the interaction among species. Furthermore, the same approach can be adapted to predict the perturbation patterns of other systems, such as food webs, social networks, and financial networks. Due to the scarcity of the exact kinetic parameters, it is essential to predict the perturbation patterns using network topology-based models. In this work, we leverage graph convolutional networks to enhance the perturbation pattern prediction. By incorporating the graph structure into the learning process, graph neural networks retain a state that gathering information from neighbors of particular nodes. Graph neural networks give predictions of impact patterns in biological networks and serve as a complement component of the topology knowledge-based model. Our experiments on 87 biological models outperform the pure network topology-based model and reveal that learning methods are adaptive and beneficial for biological perturbation pattern prediction. Our results demonstrate that the scaled impact matrix prediction predicts the perturbation patterns with 70% and 73% of accuracies using the full graph model and sparse graph model respectively. We gain 7% and 10% improvements on perturbation pattern prediction accordingly comparing to the original distance model (63%). Our findings unseal that sparse graph model outperforms the full graph model for heterogeneous networks. The most significant difference between sparse and full graph model is that the former has a specific pooling matrix according to the feature matrix of each biological network.

Our results raise several open questions: How to apply this method to predict the perturbation patterns of other systems? How to improve our method enabling high prediction accuracy? What higher-order network characteristics (such as communities, degree correlations) affect the accuracy of our prediction?
